# The immunometabolic mechanisms and therapeutic targets of metabolic dysfunction-associated steatohepatitis

**DOI:** 10.3389/fmed.2026.1801906

**Published:** 2026-05-20

**Authors:** Jiang Yu, Yong Peng

**Affiliations:** Department of General Surgery, The Second Clinical Medical College, North Sichuan Medical College, Beijing Anzhen Nanchong Hospital of Capital Medical University & Nanchong Central Hospital, Nanchong, China

**Keywords:** immunometabolism, macrophage, metabolic dysfunction-associated steatohepatitis, nuclear receptor, therapeutic targets

## Abstract

**Background:**

Metabolic dysfunction-Associated Steatohepatitis (MASH) is a progressive subtype of Metabolic dysfunction-Associated Steatotic Liver Disease (MASLD) characterized by hepatic steatosis, inflammation, hepatocellular injury, and fibrosis, which may evolve to cirrhosis and hepatocellular carcinoma. Despite its growing global burden, no widely approved pharmacotherapy is available, highlighting the need to elucidate immunometabolic mechanisms and identify effective therapeutic targets.

**Content:**

This review summarizes the epidemiology and clinical features of MASH and focuses on key pathogenic pathways, including insulin resistance, lipotoxicity, mitochondrial dysfunction, and gut-liver axis disturbance. Immune dysregulation mediated by Kupffer cell activation, macrophage polarization, inflammasome signaling, and cytokine networks is discussed in depth. The critical role of immunometabolic crosstalk in disease progression is emphasized. Current and emerging therapeutic targets—such as PPARs, FXR, THR-β, the GLP-1/FGF21 axis, DGAT2, and CCR2/CCR5—are systematically reviewed, together with advances in oligonucleotide therapy, cell-based interventions, and combination strategies.

**Conclusion:**

MASH results from the complex coupling of metabolic imbalance and immune-driven inflammation, making single-target therapy insufficient. Precision stratification based on immunometabolic networks and multi-target interventions represent promising directions for future drug development and individualized treatment.

## 1 Clinical and epidemiological overview of metabolic dysfunction-associated steatohepatitis

### 1.1 MASH: a rising epidemic at the nexus of metabolism and immunity

Steatotic liver disease (SLD) is a group of disorders characterized by abnormal intrahepatic lipid accumulation (hepatic steatosis), encompassing metabolic dysfunction-associated steatotic liver disease (MASLD), alcohol-related liver disease (ALD), A new classification for the overlap between MASLD and ALD (MetALD), and other rare causes of hepatic steatosis (1). As the inflammatory and progressive subtype of MASLD, the global prevalence of metabolic dysfunction-Associated steatohepatitis (MASH) is continuously rising in parallel with the epidemics of obesity, type 2 diabetes, and metabolic syndrome (2, 3). Unlike ALD, MASH lacks behavioral modification interventions comparable to alcohol abstinence, resulting in a greater unmet need for pharmacotherapy (4). Moreover, the complex immunometabolic crosstalk mechanisms underlying MASH have become a central focus of drug development (5). Therefore, this review focuses on the immunometabolic landscape of MASH to address the most pressing therapeutic challenges in this field.

### 1.2 Definition and diagnostic criteria of MASH

MASH is a progressive subtype of MASLD, histologically defined by steatosis, lobular inflammation, hepatocellular ballooning, and variable fibrosis. Unlike simple steatosis, MASH is an active necroinflammatory disease that can advance to cirrhosis and hepatocellular carcinoma (HCC). Fibrosis stage is the strongest prognostic determinant of liver-related outcomes (6, 7). MASH is closely associated with metabolic comorbidities, including obesity, type 2 diabetes, dyslipidemia, and metabolic syndrome. Insulin resistance plays a pivotal role in its pathogenesis by promoting hepatic lipid accumulation and increasing hepatocyte susceptibility to lipotoxic injury. Genetic predisposition and environmental factors also critically modulate disease susceptibility and progression (8–10).

### 1.3 Epidemiological features and disease burden

MASH has become a major global health challenge, with its prevalence rising in parallel with obesity, type 2 diabetes, and metabolic syndrome (6, 11). The increasing incidence of MASH closely mirrors the growing burden of metabolic disorders, with most patients exhibiting insulin resistance and features of metabolic syndrome (11, 12). Longitudinal studies indicate that approximately 20% of patients with MASH may progress to cirrhosis, markedly increasing the risk of hepatic decompensation, hepatocellular carcinoma, and liver-related mortality. Moreover, cardiovascular disease remains the principal cause of death in this population (6, 13, 14). The absence of widely approved pharmacological therapies further complicates disease management and amplifies the public health impact of MASH (11, 15).

### 1.4 Clinical manifestations and risk of disease progression

Clinically, early-stage MASH is often asymptomatic and detected incidentally. When symptoms occur, they are typically non-specific, including fatigue or right upper quadrant discomfort. Insulin resistance, type 2 diabetes, obesity, and dyslipidemia represent the most prevalent clinical associations, with familial clustering supporting a role for genetic susceptibility (9, 13, 16). The natural history of MASH is heterogeneous: approximately 15%−20% of patients progress to advanced fibrosis and cirrhosis over years to decades. Among histological parameters, fibrosis stage is the strongest predictor of long-term outcomes. Once cirrhosis develops, risks of hepatic decompensation, HCC, and liver-related mortality increase substantially (6, 8, 16). Disease progression is modulated by necroinflammation severity, metabolic comorbidities, genetic background, and lifestyle behaviors (6, 17, 18). Taken together, MASH is a progressive liver disorder tightly linked to systemic metabolic dysfunction. Its escalating prevalence underscores the urgent need for effective therapeutic strategies.

## 2 Pathogenesis of metabolic dysfunction-associated steatohepatitis

### 2.1 Metabolic dysregulation in the pathogenesis of MASH

**Figure 1:**
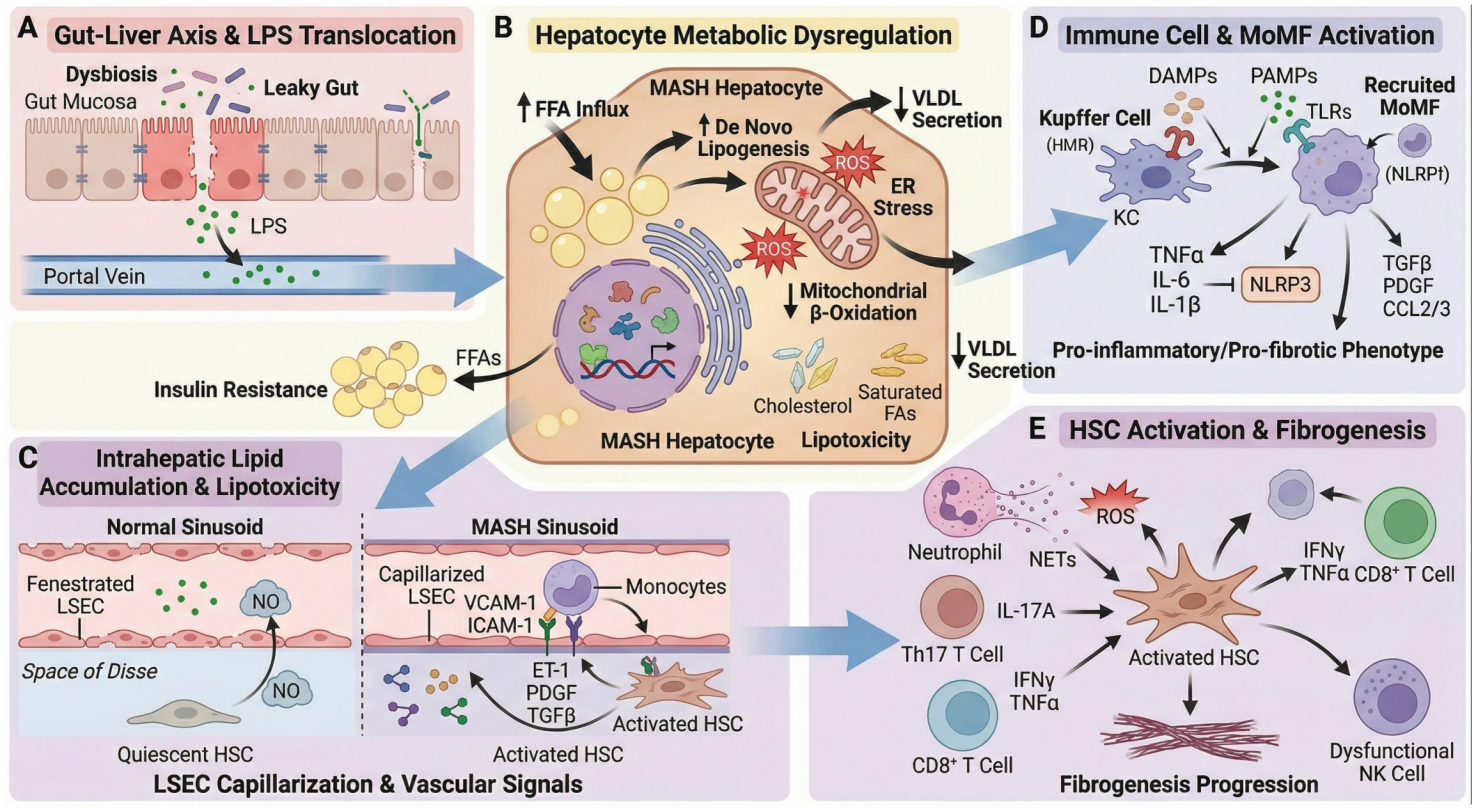
Schematic diagram of the pathogenesis of MASH.

MASH is a multifactorial metabolic liver disorder characterized by hepatic steatosis, inflammation, hepatocellular injury, and variable degrees of fibrosis. Central to MASH pathogenesis is metabolic dysregulation of lipid/glucose metabolism, insulin resistance, and systemic abnormalities (Figure 1). The earliest hallmark, hepatic steatosis, results from an imbalance between lipid acquisition and disposal in hepatocytes. This disequilibrium is driven by increased influx of free fatty acids (FFAs) derived from adipose tissue lipolysis, enhanced *de novo* lipogenesis, impaired mitochondrial β-oxidation, and reduced very-low-density lipoprotein (VLDL) secretion. Insulin resistance plays a pivotal role in this process by stimulating adipose lipolysis and hepatic lipogenesis, thereby promoting intrahepatic lipid accumulation (19). Accumulation of toxic lipid intermediates—including saturated fatty acids, free cholesterol, and ceramides—leads to lipotoxicity, which induces hepatocyte injury through oxidative stress, mitochondrial dysfunction, and endoplasmic reticulum (ER) stress. These cellular insults trigger apoptotic and necrotic pathways that represent critical events in the transition from simple steatosis to MASH (20, 21). In particular, disturbed cholesterol homeostasis has emerged as a key driver of MASH, with excess hepatic cholesterol promoting oxidative injury, inflammasome activation, and inflammatory signaling (22, 23). Dysregulation of nuclear receptors, notably peroxisome proliferator-activated receptors (PPARs) and the farnesoid X receptor (FXR), further contributes to metabolic disturbances in MASH. PPARα governs fatty acid oxidation, whereas PPARγ regulates adipogenesis and lipogenesis; aberrant activity of these receptors has been implicated in hepatic lipid overload and inflammatory responses (24). FXR orchestrates bile acid synthesis as well as glucose and lipid metabolism, and its impairment exacerbates steatosis, insulin resistance, and hepatic inflammation (19). Accordingly, pharmacological modulation of these nuclear receptors has shown therapeutic potential in restoring metabolic homeostasis (25). The gut-liver axis represents an additional determinant of metabolic dysregulation. Increased intestinal permeability and dysbiosis facilitate translocation of bacterial products such as lipopolysaccharide (LPS) into the portal circulation, activating hepatic innate immunity and disrupting metabolic signaling (19, 26). This gut-derived endotoxemia amplifies insulin resistance and promotes hepatic steatosis and inflammatory injury. Collectively, metabolic dysregulation in MASH reflects a complex interplay among insulin resistance, lipid accumulation, lipotoxic stress, nuclear receptor dysfunction, and gut-liver axis perturbation. These interconnected abnormalities initiate and perpetuate hepatocellular injury, inflammation, and fibrogenesis, providing the mechanistic foundation for MASH pathogenesis.

### 2.2 Immune system abnormalities and inflammatory responses

Inflammation defines MASH and drives its progression from simple steatosis to steatohepatitis and fibrosis. The hepatic immune system—especially innate immune cells—creates the inflammatory microenvironment that causes hepatocellular injury. Kupffer cells (KCs), the resident macrophages of the liver, play a central role in initiating and sustaining inflammation in MASH. In response to lipotoxic hepatocyte damage and gut-derived microbial signals, Kupffer cells activate and secrete pro-inflammatory cytokines (e.g., TNF-α, IL-6) and chemokines that recruit circulating monocytes and neutrophils to the liver (19, 27). Recruited monocyte-derived macrophages further amplify inflammatory cascades and promote fibrogenesis through paracrine activation of hepatic stellate cells (HSCs) (28, 29). Beyond macrophages, additional innate immune populations—such as dendritic cells (DCs), neutrophils, and innate lymphoid cells—contribute to the inflammatory response. These cells recognize DAMPs and PAMPs via pattern recognition receptors such as TLRs, activating the NLRP3 inflammasome and subsequent IL-1β production, which exacerbates hepatic inflammation and cell death (30, 31). Platelets have also emerged as active participants in MASH by facilitating immune cell recruitment and activation and by directly stimulating HSC activation, thereby providing a mechanistic link between inflammation and fibrosis (32). Macrophage functional heterogeneity is increasingly recognized as critical in MASH pathogenesis. Although a predominance of pro-inflammatory M1-like phenotypes has been described in early disease stages, producing mediators that aggravate hepatocyte injury, this binary M1/M2 classification oversimplifies the dynamic spectrum of macrophage states observed *in vivo*. Reparative and pro-resolving macrophage subsets participate in tissue remodeling and fibrosis regression, and therapeutic strategies aimed at reprogramming macrophage function rather than strict polarization are currently under investigation (29, 33). Overall, immune dysregulation in MASH involves complex crosstalk among resident and recruited immune cells, hepatocytes, and non-parenchymal cells, leading to persistent inflammation and progressive liver injury.

### 2.3 Interactions between immune regulation and metabolic imbalance

The pathogenesis of MASH is increasingly understood as a dynamic interplay between metabolic dysfunction and immune dysregulation, conceptualized as immunometabolism. Metabolic disturbances such as insulin resistance and lipotoxicity not only induce hepatocellular injury but also profoundly reshape immune cell function and inflammatory responses. Lipotoxic hepatocytes release damage-associated molecular patterns (DAMPs) and extracellular vesicles, which activate innate immune cells, including Kupffer cells and recruited macrophages, thereby mechanistically linking metabolic stress to hepatic inflammation (26, 34). These activated immune cells subsequently secrete cytokines and chemokines that further aggravate insulin resistance and disrupt lipid handling, establishing a self-perpetuating vicious cycle between metabolic and immune dysfunction (11, 35). Hormonal and metabolic regulators, particularly glucagon-like peptide-1 (GLP-1) and fibroblast growth factor 21 (FGF21), exert dual actions on metabolism and immunity. GLP-1 receptor agonists enhance insulin sensitivity and reduce hepatic steatosis, whereas FGF21 analogs attenuate inflammatory activity and fibrogenesis, underscoring the therapeutic potential of targeting metabolic–immune crosstalk (36, 37). Macrophage immunometabolism has emerged as a pivotal determinant of MASH progression. Metabolic reprogramming dictates macrophage phenotype and effector function: a shift toward glycolytic metabolism favors pro-inflammatory states, whereas oxidative phosphorylation supports pro-resolving and reparative programs. Accordingly, modulation of macrophage metabolic pathways represents a promising strategy to restrain hepatic inflammation and fibrosis (33, 38). In addition, nuclear receptors such as PPARs and FXR integrate metabolic and immune signaling networks, coordinating lipid homeostasis with inflammatory responses (25, 35). The gut microbiota further shapes this axis through the production of bioactive metabolites that influence hepatic metabolism and immune activation (26). Overall, the reciprocal interaction between immune regulation and metabolic imbalance forms a complex network that drives the initiation and progression of MASH. Therapeutic strategies aimed at reprogramming this immunometabolic axis offer a rational avenue for effective disease management.

### 2.4 The neglected portal cells: LSECs capillarization and pathological vascular secretory signals

Hepatic sinusoidal endothelial cells (LSECs) serve as the crucial interface between blood flow and liver parenchyma. Under healthy conditions, LSECs maintain a unique fenestrated phenotype, allowing for free exchange of large molecules and continuously generating stable vascular secretory signals such as nitric oxide, which maintains hepatic sinusoidal dilation and the quiescence of HSCs (39). In MASH, chronic metabolic stress triggers “capillarization” in LSEC: the fenestrations disappear, the basement membrane forms, and the secretion profile shifts from vasculoprotective to pro-inflammatory and fibrotic. The capillarized LSECs downregulates eNOS, reduces nitric oxide, leading to intrahepatic vasoconstriction and hypoxia; at the same time, it upregulates VCAM-1/ICAM-1, recruits monocytes/macrophages, and secretes factors such as endothelin-1, PDGF, and TGFβ, directly activating HSCs (40). This pathological vascular secretion signal constitutes a crucial link between metabolic stress and fibrosis. Recent studies by Hu et al. have shown that selective targeting of LSECs and perivascular cells with ROCK2 can reverse capillarization and treat established fibrosis, highlighting the potential of LSECs as a therapeutic target for MASH (41).

### 2.5 Immune cells drive the activation of HSCs and the progression of fibrosis

In the MASH model, innate and adaptive immune cells infiltrating the liver secrete a range of cytokines, chemokines, and growth factors, which in turn directly or indirectly activate HSCs, thereby driving the progression of fibrosis (42). Macrophages are the key immune cell population that regulates the activation of HSCs. The infiltrating monocyte-derived macrophages (MoMFs) and the resident Kupffer cells (KCs) both polarize toward a pro-fibrotic phenotype. They directly activate HSCs by secreting TGF-β, PDGF, IL-1β and chemokines (such as CCL2/3), thereby promoting their proliferation and collagen deposition (42, 43). Furthermore, the T cell subsets exert complex and sometimes opposite effects: CD8+ T cells activate HSCs by secreting IFN-γ and TNF-α; while in CD4+ T cells, Th17 cells secrete IL-17A to promote HSC activation, and Treg cells may inhibit fibrosis by secreting IL-10 (44). Natural killer cells (NK cells) possess the anti-fibrotic potential to eliminate activated HSCs and senescent hepatic stellate cells. However, their cytotoxic function is impaired in MASH (45). Dendritic cells (DCs) can acquire a pro-inflammatory phenotype in the MASH liver microenvironment and contribute to fibrosis by promoting T cell responses and recruiting monocytes (46). Neutrophils, on the other hand, directly activate HSCs and induce fibrosis by releasing neutrophil extracellular traps (NETs), elastase, and reactive oxygen species (ROS) (47). Therefore, a thorough understanding of the interaction network between immune cells and HSCs is of vital importance for the development of MASH anti-fibrosis treatment strategies based on immune metabolic regulation.

MASH progression is mediated by a complex interplay of metabolic dysregulation, gut-liver axis perturbation, immune activation, and vascular dysfunction. Intestinal dysbiosis and LPS translocation exacerbate hepatic insulin resistance and lipid accumulation, triggering lipotoxicity, oxidative stress, and hepatocellular injury. LSEC capillarization and pathological vascular signaling promote monocyte recruitment and HSC activation. Kupffer cells and recruited MoMFs are activated by DAMPs/PAMPs, secreting pro-inflammatory and pro-fibrotic mediators that amplify inflammation. Multiple immune cell subsets further drive HSC activation and progressive hepatic fibrosis.

## 3 Immunometabolic therapeutic targets for MASH

### 3.1 Mainstream metabolic therapeutic targets and mechanisms of action

**Figure 2:**
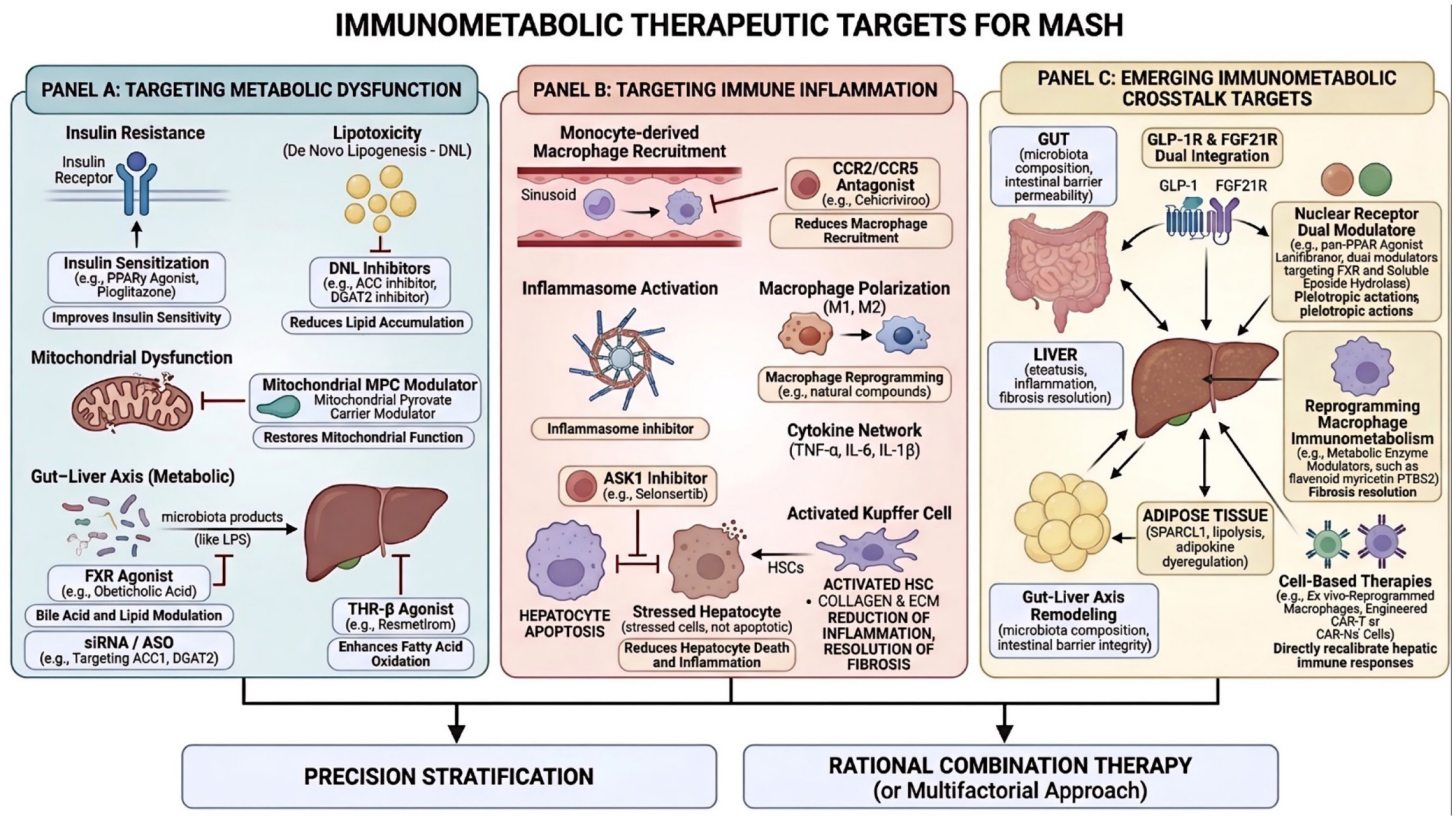
Therapeutic targets in the pathogenesis of MASH.

MASH is a multifaceted metabolic liver disease characterized by hepatic steatosis, inflammation, hepatocellular injury, and varying degrees of fibrosis. The mainstream therapeutic targets for MASH are shown in Figure 2. Hepatic lipid overload resulting from insulin resistance and enhanced *de novo* lipogenesis induces lipotoxic stress and mitochondrial dysfunction, while increased intestinal permeability and microbiota-derived products further aggravate hepatocellular injury through the gut-liver axis. These metabolic insults trigger activation of Kupffer cells and recruitment of monocyte-derived macrophages, leading to inflammasome activation, dysregulated macrophage phenotypes, and excessive production of pro-inflammatory cytokines such as TNF-α, IL-6, and IL-1β. The ensuing immunometabolic vicious cycle promotes hepatocyte death, hepatic stellate cell activation, and progressive fibrosis. Based on this framework, pursued therapeutic targets include PPARs, FXR, THR-β, DGAT2, CCR2/CCR5, GLP-1/FGF21 regulators, and siRNA/cell-based strategies, with precision stratification plus rational combination therapy increasingly viewed as a promising approach to disrupt this pathogenic network. Metabolic abnormalities involving glucose, lipid, and bile acid homeostasis constitute the central therapeutic focus in MASH. Current pharmacological strategies aim to modulate these pathways to halt or reverse disease progression. Peroxisome proliferator-activated receptors (PPARs) are nuclear receptors that coordinate lipid metabolism, glucose homeostasis, and inflammatory responses. Agonists targeting PPAR-α, PPAR-γ, or multiple isoforms have demonstrated the ability to improve insulin sensitivity, reduce hepatic steatosis, and attenuate inflammation and fibrosis. Pioglitazone, a PPAR-γ agonist, has shown significant histological benefit in biopsy-proven MASH, although weight gain and fluid retention remain clinical concerns (48). Novel selective modulators such as MSDC-0602K, which targets the mitochondrial pyruvate carrier, have been developed to improve metabolic profiles with potentially fewer adverse effects (49). Moreover, the pan-PPAR agonist lanifibranor has yielded encouraging phase 2 results, exerting pleiotropic metabolic and anti-inflammatory actions through simultaneous activation of multiple PPAR isoforms (50). Thyroid hormone receptor-β (THR-β) is critical for hepatic lipid oxidation and energy expenditure. Resmetirom, a selective THR-β agonist, has demonstrated robust reductions in hepatic fat and histological improvement in phase 2/3 trials, with a favorable safety profile (51). Mechanistically, THR-β activation enhances fatty acid oxidation and mitochondrial function, contributing to mitigation of steatosis and fibrosis. Fibroblast growth factor 21 and its analogs regulate systemic glucose and lipid metabolism while exerting anti-inflammatory and anti-fibrotic effects. Agents such as pegozafermin have shown improvements in steatosis and fibrosis-related biomarkers in early clinical studies (37, 52). Similarly, GLP-1 receptor agonists, originally developed for type 2 diabetes, improve insulin sensitivity and reduce hepatic fat. Trials with liraglutide and semaglutide have reported meaningful histological responses in MASH (36, 53), highlighting the therapeutic value of incretin-based strategies. Targeting key enzymes in lipid synthesis represents another approach to reduce lipotoxicity. Acetyl-CoA carboxylase inhibitors and diacylglycerol acyltransferase 2 (DGAT2) inhibitors have demonstrated reductions in hepatic triglyceride accumulation and signals of antifibrotic activity in preclinical and early-phase studies (54). These agents directly address the lipid burden that drives hepatocellular injury. The farnesoid X receptor is a master regulator of bile acid, lipid, and glucose metabolism. FXR agonists such as obeticholic acid have improved fibrosis endpoints in clinical trials, although pruritus and dyslipidemia necessitate careful management (55). Dual modulators targeting FXR together with soluble epoxide hydrolase have shown enhanced antifibrotic efficacy in experimental models (56). Modulation of cholesterol metabolism, mitochondrial function, and oxidative stress has also been explored. Vitamin E has demonstrated benefit in selected non-diabetic patients, whereas statins primarily address cardiovascular risk with largely neutral hepatic histological effects (48, 57). Given the multifactorial nature of MASH, combination regimens targeting complementary pathways are increasingly advocated to maximize efficacy while limiting adverse effects (58). Collectively, therapies directed at metabolic dysregulation remain the cornerstone of current drug development, and ongoing trials will further define their role in clinical practice.

### 3.2 Mainstream immunomodulatory therapeutic targets and mechanisms of action

Inflammation is a central hallmark of MASH and a major driver of progression from simple steatosis to advanced fibrosis and cirrhosis. Hepatic immune cells—including macrophages, neutrophils, and lymphocyte subsets—coordinate the inflammatory microenvironment, making immune pathways attractive therapeutic targets to mitigate liver injury and fibrogenesis. Resident Kupffer cells and infiltrating monocyte-derived macrophages drive MASH by producing pro-inflammatory mediators (TNF-α, IL-6, IL-1β). Therefore, therapeutic strategies targeting macrophage recruitment, activation, and functional reprogramming have been intensively explored. CCR2/CCR5 antagonists reduce hepatic macrophage infiltration and inflammation, and the dual inhibitor cenicriviroc showed antifibrotic signals in phase 2 studies, though subsequent trials underscored the need for optimized patient selection and combination regimens (11, 34). In parallel, experimental studies of natural compounds capable of reshaping macrophage immunometabolism have shown anti-inflammatory effects in preclinical models, yet their translational relevance requires rigorous clinical validation (33, 38). Systemic and intestinal immune responses critically influence hepatic inflammation. Approaches designed to modulate gut immunity—such as oral anti-CD3 antibodies, anti-lipopolysaccharide antibodies, and bioactive dietary extracts—have been proposed to attenuate systemic inflammatory tone without inducing broad immunosuppression (59). Although these strategies aim to restore mucosal immune tolerance and reduce pro-inflammatory signaling, clinical evidence is limited; platelets promote MASH by recruiting leukocytes and activating hepatic stellate cells, suggesting antiplatelet interventions could interrupt the inflammation-fibrosis axis (32). Moreover, adipose tissue-derived mediators such as secreted protein acidic and rich in cysteine-like protein 1 (SPARCL1) have been implicated in hepatic immune activation, representing additional targets for intervention (60). Nuclear receptor agonists, including FXR and PPAR ligands, exert indirect immunoregulatory actions by modulating cytokine production and immune cell phenotypes, thereby bridging metabolic and inflammatory pathways (11, 61). Apoptosis signal-regulating kinase 1 (ASK1) inhibitors, exemplified by selonsertib, were developed to reduce hepatocyte apoptosis and inflammation. However, large phase 3 trials failed to meet primary fibrosis endpoints, underscoring the challenges of single-pathway targeting in MASH (62). Collectively, emerging immunotherapies seek to restrain immune cell activation and recruitment, modulate cytokine networks, and promote resolution of inflammation. While several approaches have shown encouraging mechanistic rationale, robust clinical efficacy has yet to be established, and combination strategies are likely required to achieve durable histological benefit.

### 3.3 Emerging immunometabolic crosstalk therapeutic targets

The intricate interaction between metabolic dysfunction and immune activation in MASH has established immunometabolic crosstalk as an emerging therapeutic frontier. This concept seeks to target pathways that concurrently regulate metabolic homeostasis and immune responses, thereby achieving synergistic disease modification. The glucagon-like peptide-1 and fibroblast growth factor 21 pathways exemplify this dual regulation. GLP-1 receptor agonists improve glycometabolic control while exerting anti-inflammatory effects on hepatic and extrahepatic immune cells, leading to reductions in steatosis and inflammatory activity. FGF21 analogs complement these actions by enhancing lipid oxidation and suppressing profibrotic signaling (36, 37). Combination strategies integrating incretin-based agents with other metabolic modulators are currently under active clinical evaluation. Reprogramming macrophage metabolism represents a compelling approach to reshape hepatic inflammation. Although a shift from pro-inflammatory toward pro-resolving phenotypes has been proposed as beneficial, the *in vivo* spectrum of macrophage states is highly plastic and context-dependent. Small molecules capable of modulating key metabolic enzymes have shown anti-inflammatory effects in experimental models; for instance, the flavonoid myricetin inhibits prostaglandin-endoperoxide synthase 2 (PTGS2) and attenuates macrophage-mediated injury (29, 33, 63). Nevertheless, translation of such agents requires stringent pharmacokinetic and safety assessment. The gut-liver axis integrates microbial metabolites with host immunity and metabolism. Strategies aimed at remodeling microbiota composition, enhancing intestinal barrier integrity, or interrupting extracellular vesicle-mediated communication offer innovative opportunities to reduce systemic and hepatic inflammation while restoring metabolic balance (26). Nuclear receptors including PPARs and FXR function as central nodes linking metabolic and immune networks. Dual or multi-target modulators have demonstrated superior efficacy compared with single-pathway interventions in preclinical studies (35, 56). In addition, exploratory cell therapies—such as *ex vivo*-reprogrammed macrophages and engineered CAR-T or CAR-NK cells—are being investigated to directly recalibrate hepatic immune responses and facilitate fibrosis resolution, although these approaches remain at an early translational stage (34). In summary, therapeutic strategies directed at immunometabolic crosstalk provide a rational framework to address the multifactorial nature of MASH. Future success will likely depend on precision stratification of patients and rational combination regimens capable of simultaneously correcting metabolic dysfunction and immune-mediated inflammation.

This figure summarizes the three main categories of targets in MASH therapy, among which precise stratification and rational combination therapy constitute the overall clinical strategy. Figure A: Metabolic dysfunction targets, including insulin sensitizers, fatogenesis inhibitors, mitochondrial regulators, FXR/THR-β agonists, and nucleic acid therapies. Figure B: Immune-inflammatory targets, covering macrophage recruitment inhibitors, inflammasome blockers, macrophage reprogramming agents, cytokine regulators, and ASK1 inhibitors. Figure C: Emerging immune-metabolic interaction targets, including GLP-1/FGF21 dual agonists, nuclear receptor dual regulators, macrophage immune-metabolic reprogramming, cell therapies, and intestinal-liver axis remodeling.

## 4 Clinical treatment strategies and drug development progress

### 4.1 Currently available therapies and evidence appraisal

MASH remains a major unmet medical need, and lifestyle modification—caloric restriction, dietary optimization, and increased physical activity—continues to represent the foundation of management. Pharmacotherapy is generally considered for patients who fail to achieve sustained weight loss or who present with significant fibrosis and metabolic comorbidities (Table 1). Pioglitazone has provided the most consistent histological evidence among repurposed agents, improving steatosis, inflammation, and hepatocellular ballooning, with signals toward fibrosis benefit in selected trials. Its use, however, is tempered by weight gain, edema, and long-term safety considerations (48). Vitamin E demonstrated improvement in necroinflammatory activity in non-diabetic patients, yet uncertainties regarding long-term cardiovascular and oncologic safety restrict universal adoption (48, 57). Current guidelines therefore recommend these agents only for carefully selected individuals with biopsy-proven disease (64). Metformin has not shown reproducible histological efficacy and is not recommended specifically for MASH (65). Statins and fibrates remain essential for cardiovascular risk reduction but confer limited direct hepatic benefit (57). Pentoxifylline, ursodeoxycholic acid, and renin–angiotensin system blockers have produced heterogeneous results and lack robust evidence for routine use (12, 57). Resmetirom and semaglutide are two drugs approved by the US Food and Drug Administration (FDA) for the treatment of MASH. Resmetirom is a THR-β agonist that directly promotes liver fatty acid β-oxidation, alleviates lipid toxicity, and improves lipid profiles by enhancing mitochondrial biosynthesis and autophagy. It was approved for use in MASH patients with moderate to advanced fibrosis in 2024. The phase III clinical trial demonstrated that Resmetirom can significantly reduce liver fat content, liver enzyme levels, low-density lipoprotein cholesterol levels, and alleviate fibrosis. Semaglutide is a long-acting GLP-1 receptor agonist. Due to the absence of GLP-1 receptors in the liver, it has no direct liver activity. Its main mechanism of action is to indirectly provide liver benefits by improving overall metabolism (promoting insulin secretion, reducing appetite, and weight loss). In 2025, it was approved by the FDA based on its III-phase data, with the indication being MASH patients with the same fibrosis stage. As the only two approved drugs for MASH treatment at present, the optimal application strategies for using either drug alone or in combination still need to be further explored (66, 67).

**Table 1:** Summary table of investigational drugs for MASH.

Drug class	Representative agents	Key evidence/development status	Major limitations/safety concerns
PPAR agonists	Pioglitazone, elafibranor, lanifibranor, saroglitazar	Pioglitazone: histological benefit; elafibranor: phase 3 failure; lanifibranor: positive phase 2; saroglitazar: approved in India	Weight gain, edema, class heterogeneity
FXR agonists	Obeticholic acid	Phase 3 fibrosis benefit but not fully approved	Pruritus, dyslipidemia
THR-β agonists	Resmetirom	FDA approved (2024)	Generally well-tolerated
GLP-1 receptor agonists	Liraglutide, semaglutide	Semaglutide: FDA approved (2025)	Gastrointestinal effects
FGF21 analogs	Pegozafermin, efruxifermin	Early trials: improved fibrosis/metabolic markers	Confirmatory trials needed
Antioxidants	Vitamin E	Efficacy in non-diabetic patients	Long-term cardiovascular/oncologic safety concerns
Failed targeted therapies	Selonsertib, cenicriviroc	Phase 3 failure or no MASH resolution	Single-pathway limitations

### 4.2 Investigational agents and clinical trial landscape

Beyond the approved agents described above, intensive research has generated a diverse pipeline targeting metabolism, inflammation, apoptosis, and fibrosis. Obeticholic acid improved fibrosis without worsening steatohepatitis in the REGENERATE trial, yet pruritus and adverse lipid changes remain limiting factors (55, 68). Elafibranor failed to meet phase 3 endpoints despite earlier signals (61), whereas the pan-PPAR agonist lanifibranor demonstrated encouraging phase 2 efficacy across steatohepatitis and fibrosis domains (50). Cenicriviroc yielded antifibrotic signals in phase 2b but did not achieve MASH resolution (61). Selonsertib was unsuccessful in phase 3 studies, illustrating the limitations of single-pathway approaches (61). Other GLP-1 receptor agonists (e.g., liraglutide) reduce steatosis and improve histology largely through weight-loss-mediated metabolic effects (36, 62). FGF21 analogs such as pegozafermin and efruxifermin have shown improvements in lipid metabolism and fibrosis markers in early trials (37, 52). Oligonucleotide therapies targeting genes involved in lipogenesis and inflammation (69), as well as rational combination regimens (58), are being explored. Saroglitazar is approved in India with metabolic benefits reported in phase 2 studies (70). Despite these advances, no agent has yet achieved universal approval in major Western markets, reflecting the biological complexity of MASH and the challenges of histology-based endpoints.

### 4.3 Strengths and limitations of immunometabolic-targeted therapies

Therapies addressing both metabolic and immune pathways offer the theoretical advantage of modifying core drivers—insulin resistance, lipotoxicity, oxidative stress, immune activation, and fibrogenesis (69, 71). Nuclear receptor agonists offer pleiotropic metabolic and anti-inflammatory effects (34, 50); GLP-1 and FGF analogs improve systemic homeostasis with secondary hepatic benefits (36, 69); immune-directed agents targeting chemokine receptors or galectin-3 aim to restrain fibrogenic inflammation without global immunosuppression (71, 72). However, substantial limitations persist: Disease heterogeneity: variable molecular phenotypes hinder identification of universally effective agents (73). Safety/tolerability: weight gain (pioglitazone), pruritus/dyslipidemia (OCA), and gastrointestinal effects (GLP-1RAs) restrict adherence (12, 34). Trial design challenges: slow progression necessitates large, long studies with invasive endpoints (71). Targeting complexity: diverse immune cell functions require precise modulation to avoid off-target immunosuppression (5, 28). Combination therapy is therefore viewed as a rational path forward, though optimal regimens and patient-selection biomarkers remain to be defined (58). In summary, immunometabolic therapies represent a transformative yet challenging frontier. Integration of systems biology, non-invasive biomarkers, and precision stratification will be essential to translate these approaches into durable clinical benefit (71, 74).

## 5 Challenges, unmet needs, and future outlook

### 5.1 Key barriers to effective MASH therapy

MASH is a biologically heterogeneous disorder driven by the convergence of metabolic overload, immune dysregulation, and fibrogenic remodeling. This complexity constitutes the fundamental barrier to drug development, as interventions directed at a single pathway rarely achieve durable disease modification (34). Patients differ markedly in genetic susceptibility, metabolic phenotype, and dominant injury drivers, leading to highly variable therapeutic responses (33, 72). Weight reduction remains the most effective intervention, yet long-term adherence is poor in routine practice (75). Consequently, most patients require pharmacological support, but currently available agents provide only partial histological benefit and are constrained by safety concerns (76, 77). The reliance on liver biopsy as the reference standard introduces sampling variability and limits patient acceptance (78). Slow fibrosis dynamics necessitate large, long trials with composite histological endpoints, contributing to the high attrition rate of candidate drugs (15). Disease progression involves coordinated interactions among hepatocytes, Kupffer cells, infiltrating macrophages, stellate cells, and the gut-liver axis (5, 29). Therapeutic modulation of immune pathways is particularly challenging because excessive suppression may impair host defense, whereas incomplete modulation fails to halt fibrogenesis (79). Collectively, multifactorial pathogenesis, patient heterogeneity, and the absence of reliable non-invasive monitoring tools remain the major unmet needs in MASH management.

### 5.2 Biomarkers and the road toward precision medicine

The transition from empirical therapy to precision medicine depends on robust biomarkers that reflect disease activity, fibrogenic risk, and treatment response. Metabolomic profiling has identified early disturbances in cholesterol and bile-acid pathways preceding histological injury (22). Extracellular vesicles and microRNAs are emerging as indicators of immune activation and fibrotic remodeling, offering real-time insight into inter-organ communication (26). ASGPR-targeted probes enable functional assessment of hepatocyte mass in preclinical models and may support translational monitoring (80). Integration of genomic risk variants, metabolic signatures, and immune phenotypes is expected to define treatable traits and guide therapy selection (72, 74). Systems-biology frameworks linking multi-omics to clinical outcomes will be essential for adaptive trial designs and enrichment strategies (71).

### 5.3 Future development of immunometabolic targets

Therapeutic innovation is increasingly oriented toward immunometabolic hubs that couple lipid handling with immune regulation. Nuclear receptor networks: FXR and PPAR modulators exert pleiotropic control over metabolism and inflammation, yet next-generation agents with improved selectivity and safety are required (34, 55, 61). Endocrine regulators: FGF21 analogs and incretin-based therapies provide complementary metabolic and anti-inflammatory actions, supporting rational combination with nuclear receptor agonists (25, 37). Gene-directed approaches: Antisense and siRNA platforms (e.g., PNPLA3 silencing) enable precision targeting of genetic drivers with potentially favorable off-target profiles (69). Macrophage reprogramming: Modulation of macrophage immunometabolism to restore M2-like homeostasis is a promising strategy, supported by both pharmacologic and nutraceutical interventions. Multi-target small molecules. Dual FXR/sEH modulators exemplify efforts to integrate antifibrotic and metabolic effects within a single scaffold (56). Advanced modalities: Restorative macrophages and CAR-T-based approaches are being explored for active fibrosis resolution (34). Combination therapy tailored to dominant disease drivers is likely to supersede monotherapy, while digital pathology and non-invasive biomarkers will enable response-guided treatment (61, 79).

### 5.4 From heterogeneity to molecular subtypes: key issues in MASH precision medicine

A key limitation of current MASH treatment is the lack of systematic consideration of its molecular subtypes. Emerging transcriptomic and proteomic analyses are beginning to address this gap by identifying patient subgroups characterized by distinct pathological drivers. The latest study, by integrating multi-omics analyses (liver proteome, phosphorylated proteome, whole-genome sequencing, serum and urine metabolome), classified MASH into three clinical molecular subtypes in the Chinese population: metabolically active type (lipid metabolism disorder), high-risk liver cirrhosis type (significantly activated fibrosis signaling), and high-risk primary liver cancer type (activation of proliferation or carcinogenesis pathways) (81). Multimodal studies have revealed extensive dysregulation of genes related to lipid metabolism, inflammation, and fibrosis, differential expression of proteins related to disease progression, as well as significant changes in various lipid molecules such as ceramides, sphingolipids, phospholipids, and bile acids (82, 83). These subtypes exhibit varying responses to pathway-targeted drugs in preclinical models. This molecular stratification provides a crucial background for understanding why single-targeted treatments often fail in unselected MASH populations: drugs targeting lipotoxicity may be effective for metabolic-driven subtypes, but may be ineffective or even harmful for patients with fibrosis or proliferative signaling as the main drivers (81, 82). Therefore, future trial designs should incorporate baseline multi-omics analysis to achieve subtype-enrichment strategies, adaptive allocation, and retrospective analysis of different treatment effects.

## 6 Conclusion

MASH represents a prototypical immunometabolic disease in which metabolic stress and innate immunity converge to drive fibrogenesis. Although no universally approved therapy is yet available, rapid advances in molecular phenotyping, biomarker science, and targeted therapeutics are reshaping the field. Future success will depend on: (1) mechanism-based patient stratification, (2) non-invasive surrogate endpoints, (3) rational combination regimens, and (4) integration of systems biology with real-world evidence. Such strategies are expected to transform MASH from a progressive disorder into a preventable and treatable condition.
